# No Cure, No Pay

**Published:** 1886-07

**Authors:** 


### NO CURE, NO PAY!

We extract the following from a late number of the Parisian *Journal D'Hygiene*, under the caption of "some medical anecdotes prior to 1790."

Among the Wisigoths, physicians stipulated to cure the patient for a certain sum, and if the patient died the physician received no pay. If he injured one by bleeding him, he was required to pay damages. If the patient were a freeman and died immediately after being bled, the physician was delivered up to the parents of the deceased to be punished to their satisfaction. On the other hand, if the deceased were a serf, the physician was exonerated by giving them another serf in his place.

A celebrated physician who had the honor of association with the Regent, upon hearing him discourse upon medicine as an experimental or conjectural art, thus terminated the discussion ; "Suppose Paris should all at once be shrouded in Egyptian darkness ; is it not true Monseigneur, that you would prefer to commit yourself to the guidance of a blind man accustomed to feel his way unerringly with his stick, rather than to one ever so clear sighted, who would out of necessity lead you altogether at random amid the impenetrable gloom!"

A renowned physician in paying his professional visits, always went into the kitchen, embraced the cooks and chief purveyors and said to them ; "my good friends, I am thankful for all the good services you renders me and my brother physicians ; without you and your art of poisoning, the faculty would soon be in the alms house.

The carriage of Guenot, a famous physician was once brought to a stand in the market place, by a jam of carriages which he found it impossible to break through, when a driver cried aloud to his comrades, "give way there, allow the physician to pass ; I know him ; it is the one who did us the service to kill Cardinal Mazarin."

A certain Swiss physician never passed his town cemetery without covering his face with his handkerchief. When asked the reason of this he replied; "it is because many of those who lie there were brought to it by my treatment, and I am always apprehensive that some of them may recognize me, and without warning take me by the throat."

